# Enhancement of biocompatibility of anodic nanotube structures on biomedical Ti–6Al–4V alloy via ultrathin TiO_2_ coatings

**DOI:** 10.3389/fbioe.2024.1515810

**Published:** 2024-12-02

**Authors:** Marcela Sepúlveda, Jan Capek, Kaushik Baishya, Jhonatan Rodriguez-Pereira, Jana Bacova, Stepanka Jelinkova, Raul Zazpe, Hanna Sopha, Tomas Rousar, Jan M. Macak

**Affiliations:** ^1^ Center of Materials and Nanotechnologies, Faculty of Chemical Technology, University of Pardubice, Pardubice, Czechia; ^2^ Central European Institute of Technology, Brno University of Technology, Brno, Czechia; ^3^ Department of Biological and Biochemical Sciences, Faculty of Chemical Technology, University of Pardubice, Pardubice, Czechia

**Keywords:** Ti-6Al-4V alloy, TiO_2_ nanotube layers, atomic layer deposition, MG-63 cells, cell growth, cell proliferation

## Abstract

This work aims to describe the effect of the surface modification of TiO_2_ nanotube (TNT) layers on Ti-6Al-4V (TiAlV) alloy by ultrathin TiO_2_ coatings prepared via Atomic Layer Deposition (ALD) on the growth of MG-63 osteoblastic cells. The TNT layers with two distinctly different inner diameters, namely ∼15 nm and ∼50 nm, were prepared via anodic oxidation of the TiAlV alloy. Flat, i.e., non-anodized, TiAlV alloy foils were used as reference substrates. Additionally, a part of the TNT layers and alloy foils was coated with ultrathin coatings of TiO_2_ by ALD. The number of TiO_2_ ALD cycles used was 1 and 5 leading to a nominal TiO_2_ thickness of ∼0.055 and ∼0.3 nm, respectively. The ultrathin TiO_2_ coating by ALD enabled to optimize the surface hydrophilicity for optimal cell growth. In addition, coatings shaded impurities of V- and F-based species (stemming from the alloy and the anodization electrolyte) that affect the biocompatibility of the tested materials while preserving the original structure and morphology. The evaluation of the biocompatibility before and after TiO_2_ ALD coating on TiAlV flat surfaces and TNT layers was carried out using MG-63 osteoblastic cells and compared after incubation for up to 96 h. The cell growth, adhesion, and proliferation of the MG-63 on TiAlV foils and TNT layers showed significant enhancement after the surface modification by TiO_2_ ALD.

## 1 Introduction

Titanium (Ti) and its alloys stand out among commercially available metallic implant biomaterials, demonstrating consistent applicability attributed to their satisfactory mechanical properties, excellent biocompatibility, and corrosion resistance (Chen and Thouas, 2015; Sarraf et al., 2021). Approximately 50% of biomedical implants are manufactured using Ti-6Al-4V (TiAlV) alloy with an α + β phase composition (Singh and Dahotre, 2007). Nevertheless, the exposure of metallic implants to highly corrosive body fluids triggers corrosion processes that may negatively impact both the biocompatibility and mechanical integrity of the implants (Oliveira et al., 2006). In addition to corrosion, metallic implants may be susceptible to other forms of degradation, including wear and tribocorrosion (Campoccia et al., 2006). These processes can lead to the release of metallic particles and/or ions, often associated with inflammatory responses and the activation of bone-resorbing cells (osteoclasts) (Vasconcelos et al., 2016; Costa et al., 2019). This avalanche of events may result in osteolysis (bone resorption) and, ultimately, implant loosening. The biological effects associated with them remain incompletely understood, and their long-term impacts cannot yet be safely predicted (Zhao and Castranova, 2011; Konttinen and Pajarinen, 2013). To prolong the effective lifetime of implant materials, it is essential to further improve the osseointegration and corrosion resistance of the exposed implant material against undesired biochemical reactions of TiAlV (Nune et al., 2018; Im et al., 2022).

Microroughness has been extensively discussed in the literature to have a beneficial effect on the osseointegration of implants (Gittens et al., 2014). This can be achieved by nanostructuring the implant’s surface, leading to improved osseointegration, corrosion resistance, and longevity of the implants (Magesh et al., 2018; Subramani et al., 2018). Several techniques can be employed to nanostructure surfaces, allowing them to mimic the morphology of living tissue (Long and Rack, 1998), including chemical etching (Klokkevold et al., 1997), ion implantation (De Maeztu et al., 2003), or anodic oxidation (Matykina et al., 2007), which are accessible to enhance and modify the surface chemistry of an implant. Among all these techniques, anodic oxidation is a simple way to modify the surface of the titanium and its alloys by the formation of TiO_2_ nanotube (TNT) layers in F-containing electrolytes (Macak and Schmuki, 2006; Tsuchiya et al., 2007). Their exceptional features include a large surface area, unique surface chemistry, and low cytotoxicity, the combination of these factors has a beneficial impact on cellular adhesion (Park et al., 2009; Roy et al., 2011). The morphology of TNT layers can be easily manipulated by adjusting both the anodizing voltage and the electrolyte composition (Macak et al., 2008; Lee et al., 2014). Nevertheless, elements from electrolytes, such as F species, are also retained within the TNT layers and cause toxicity issues (Motola et al., 2020).

Early studies on the anodization of TiAlV alloys have demonstrated the formation of TNT layers on the alloy’s surface (Macak et al., 2005; Luo et al., 2008). However, the α and β phases of the alloy have different (moderate) dissolution and nanotube formation rates in the anodization electrolyte, resulting in a thickness difference in the TNT layer formed on each phase. The β-phase, enriched in V, dissolves more easily, resulting in thinner nanotube layers than the nanotube layers obtained on the α-phase (Macak et al., 2005; Matykina et al., 2011).

Several studies have reported on the impact of nanostructured surfaces on the growth of different types of cells (Nune et al., 2018; Im et al., 2022), promoting bone-implant integration by providing more surface area for cellular adhesion. In other words, the TiAlV alloy surfaces that were first anodized, having a TNT layer on top, showed better cellular responses such as adhesion, morphology, differentiation, mineralization, and cell seeding rate compared to the bare TiAlV alloy. A recent study of TNT layers with different diameters of nanotubes formed via anodization on TiAlV alloy shows how the adhesion and differentiation of cells can be easily controlled (Filova et al., 2015). Therefore, anodization seems to be a promising technique to enhance the osseointegration of implants. However, these studies have not emphasized the negative impact of the toxicity of F- and V-species on the biocompatibility of these TNT layers. According to the literature, TiAlV alloy implants can release V-based speciesinto the body, causing adverse health effects (Szewczenko et al., 2010; Vaithilingam et al., 2016). Biologically, the role of V in living mammals, particularly in humans, remains a topic of controversy, with conflicting data regarding its biological activity and toxicity (Goc, 2006; Korbecki et al., 2015). *In vivo* studies suggest that V can accumulate in certain organs, including the liver, and lower concentrations in the kidneys, bones, and spleen (Goc, 2006). Concerning metallic implants that incorporate V, such as TiAlV alloy, Gomes et al. (2011) proposed that V-based species could potentially cause genetic damage, such as DNA breakage.

A possible solution to this problem is a shading of V and other undesirable elements that could compromise biocompatibility properties. Atomic Layer Deposition (ALD) is an effective technique to prepare homogeneous coatings with precise thickness control even in the low nm and sub-nm range (Zazpe et al., 2016). Our previous works (Motola et al., 2020; Baishya et al., 2023; Capek et al., 2024), showed that such ultrathin TiO_2_ ALD coating on top of TNT layers on Ti foils and TiAlV alloy foils results in shading of unwanted species, such as F- and V-based species, improving the biocompatibility of the materials without changing their surface morphology (Motola et al., 2020; Baishya et al., 2023; Capek et al., 2024). Several studies have described the antimicrobial activity of TiO_2_ ALD coatings, showing that modifying nanomaterial surfaces using TiO_2_ ALD coating can inhibit bacterial and yeast biofilm formation (Pessoa et al., 2017; Darwish et al., 2019; González et al., 2021). Nevertheless, a comprehensive approach with analyses that address the surface characteristics (i.e., wettability, roughness, composition, crystallinity, and biocompatibility) of smooth and anodized TiAlV alloy materials before and after ultrathin TiO_2_ coating via ALD is still lacking.

To address this gap and to understand the effects of V- and F-based species released from TiAlV alloy implants the effect of ultrathin TiO_2_ ALD coatings on the biocompatibility of TiAlV alloy foils and amorphous TNT layers on TiAlV alloy foils was evaluated in this work. At first, TNT layers with different diameters were grown on TiAlV alloy by anodization. The TiO_2_ ALD coatings were prepared on part of TNT layers using 1 and 5 ALD cycles, resulting in a nominal coating thickness of ∼0.055 and ∼0.3 nm, respectively. The cell growth and the cell mechanics, cytoskeleton structure, and adhesion on these ALD-coated compared to non-coated surfaces were evaluated using MG-63 osteoblastic cells and incubation times up to 96 h. The morphology, crystallinity, surface roughness, and wettability of the complete set of materials were assessed using SEM, XPS, static water contact angle (WCA), and Atomic Force Microscopy (AFM). Subsequently, fluorescence microscopy was employed to analyze cell growth on all evaluated materials.

## 2 Experimental details

### 2.1 Synthesis and characterization of materials

The TiAlV alloy foils (Goodfellow, 0.1 mm thick, grade 5) were cut into square pieces (1.5 × 1.5 cm^2^), degreased via sonication in acetone and isopropanol in an ultrasonic bath for 1 min, respectively, and then dried in air. TNT layers were prepared via electrochemical anodization of TiAlV foils; with the growth process taking place at room temperature in a glycerol-based electrolyte containing 50 vol% water and 0.27 M NH_4_F at 3.3 V or 15 V for 3 h, resulting in TNT layers with an inner nanotube diameter of ∼15 and ∼50 nm in average, respectively. A Pt foil was used as a counter electrode during the process. A high-voltage potentiostat (HEIDEN, EA-PSI 9200-15, Germany) attached to a digital multimeter (Keithley 2100, United States) was used for voltage control. After anodization, the TNT layers were cleaned by sonication in isopropanol for 5 min and dried in air. The TNT layers are further noted as TNT15 and TNT50 layers.

A part of the TiAlV foils and TNT layers were coated by ultrathin TiO_2_ coatings using Atomic Layer Deposition (ALD, TFS200, Beneq). The process was carried out at 300 °C using TiCl_4_ (electronic grade 99.9998%, STREM) as the Ti precursor and Milli-pore deionized water (18 MΩ) as the oxygen source. High-purity N_2_ (99.9999%) was the carrier and purging gas at a flow rate of 400 standard cubic centimeters per minute (sccm). Under these deposition conditions, one ALD growth cycle was defined by the following sequence: TiCl_4_ pulse (500 ms)–N_2_ purge (3 s)–H_2_O pulse (500 ms)–N_2_ purge (4 s). The corresponding layers are later denoted as “+ 1c TiO_2_” and “+ 5c TiO_2_”. The nominal thicknesses of 1c and 5c TiO_2_ are ∼0.055 and ∼0.3 nm, respectively, according to our previous studies (Baishya et al., 2023; Capek et al., 2024). Table 1 provides an executive summary of the TiAlV foils and TNT layers examined in this study.

**TABLE 1:** Overview of TiAlV foils and TNT layers evaluated in this study.

Structures	Surface modification	Abbreviation used in the text
Foils	-	TiAlV
	1 TiO_2_ ALD cycle	TiAlV + 1c TiO_2_
	5 TiO_2_ ALD cycle	TiAlV + 5c TiO_2_
Nanotubes	TNT layers, diameter ∼15 nm	TNT15
	TNT layers, diameter ∼15 nm + 1 TiO_2_ ALD cycle	TNT15 + 1c TiO_2_
	TNT layers, diameter ∼15 nm + 5 TiO_2_ ALD cycle	TNT15 + 5c TiO_2_
	TNT layers, diameter ∼50 nm	TNT50
	TNT layers, diameter ∼50 nm + 1 TiO_2_ ALD cycle	TNT50 + 1c TiO_2_
	TNT layers, diameter ∼50 nm + 5 TiO_2_ ALD cycle	TNT50 + 5c TiO_2_

The surface morphology of all TNT layers and foils was characterized using Scanning Electron Microscopy (FE-SEM, JEOL, JSM 7500 F). The dimensions of the TNT layers were evaluated by statistical analyses of SEM images using proprietary Nanomeasure software.

The surface chemical composition of all TiAlV foils and TNT layers was assessed by X-ray Photoelectron Spectroscopy (XPS, ESCA 2SR, Scienta Omicron) using a monochromatic Al Kα (1486.7 eV) X-ray source. The X-ray source was operated at 250 W. The binding energy scale was referenced to adventitious carbon (284.8 eV). No charging neutralizer was used during the measurements. The spectra were fitted using Shirley-type background by CasaXPS software. The quantitative analysis was performed using the elemental sensitivity factors provided by the manufacturer.

The wettability was evaluated by measuring the static water contact angle (WCA) using a Surface Energy Evaluation System device (See System E, Advex Instruments) with proprietary image analysis software. WCAs were measured at room temperature, with 3 µL droplets of DI water deposited onto the material´s surface, allowing 5 s for stabilization. The contact angles of the water droplets were determined through the tangent line analysis method. Measurements were performed at 5 different positions on each material. All results were expressed as mean ± standard deviation (SD). The contact angle measurements were carried out on as-produced materials without further cleaning or pretreatment (nanotubes after anodization and washing, ALD samples after ALD processes, and foils as delivered). This strategy was used to prevent any uncontrolled and uneven modification of surfaces, particularly to maintain the same chemistry of the surfaces for the subsequent cell tests.

The roughness of TiAlV foils and TNT layers was determined by Atomic Force Microscopy (AFM, Bruker Dimension FastScan) on an area of 5 × 5 μm^2^. Scanasyst-Air tips (fo = 70 kHz) were used.

### 2.2 Cell culture

Human osteoblast-like cells MG-63 (ATCC No. CRL-1427; doubling time, DT = 31 h) were cultured in Minimum Essential Medium (Merck) with 10% (v/v) fetal bovine serum (Gibco), 2 mmol.L^−1^ glutamine, 1% non-essential amino acids solution, and 50 μg⋅mL^-1^ penicillin/streptomycin solution (Gibco), followed by incubation in an atmosphere of 5% CO_2_ at 37 °C. Cells were proven to be mycoplasma-free, and STR analysis confirmed the origin of all cell lines.

### 2.3 Cell growth on tested materials

The square-shaped substrates were cut into round shapes with a diameter of approx. 5 mm (using sharp scissors). All tested materials were sterilized in 70% ethanol for 30 min, washed with deionized water, and dried. Then, the foils were placed on eight-well chamber slides. MG-63 cells at a density of 2 × 10^3^ cells/cm^2^ were seeded in each well of a chamber slide and cultured for 24, 48, 72, and 96 h. Cell counts were established to maintain optimal cultivation densities up to 96 h (the doubling time of the MG-63 cells was 31 h). Phalloidin-FITC and Hoechst 33,258 dyes were used to visualize actin filaments and cell nuclei, respectively. After cell culture for 24, 48, 72, and 96 h, cells were fixed with 3.7% formaldehyde (5 min; 37 °C; dark) and permeabilized with 0.1% Triton X-100 (15 min; 37 °C; dark). Then, 100 μL of phalloidin-FITC (1 µmol.L^-1^) was added and the samples were incubated at 37 °C. After 30 min of phalloidin-FITC loading, 10 μL of Hoechst 33,258 solution was added to cells. The final concentration of Hoechst 33,258 in a well was 2 μg⋅mL^-1^. After 10 min, the cells were washed twice with phosphate-buffered saline (37 °C). Actin filaments (FITC filter, 480/30 nm) and cell nuclei (DAPI filter, 375/28 nm) were observed with an Eclipse 80i fluorescence microscope (Nikon, Japan). The number of cells grown on the surface was counted from at least 35 fields of view using NIS-Elements AR (Nikon, Japan). All experiments were performed at least three times independently. The number of cell nuclei was related to 1 mm^2^ and expressed as mean ± standard error of the mean (SEM) taken from fluorescence images. Quantitative analysis of cells' elongation on tested samples was provided using NIS-Elements AR (Nikon, Japan).

### 2.4 Statistics

All cellular experiments were repeated at least three times independently. The number of estimated fields of view was (n = 35). The results are expressed as (mean ± SD). Statistical significance was analyzed after normality testing using a one-way ANOVA test followed by the Bonferroni posttest (OriginPro 9.0.0, United States) to compare results with each other at a significance level *p = 0.05*.

## 3 Results and discussion

### 3.1 Surface, structure, and composition characteristics

**FIGURE 1:**
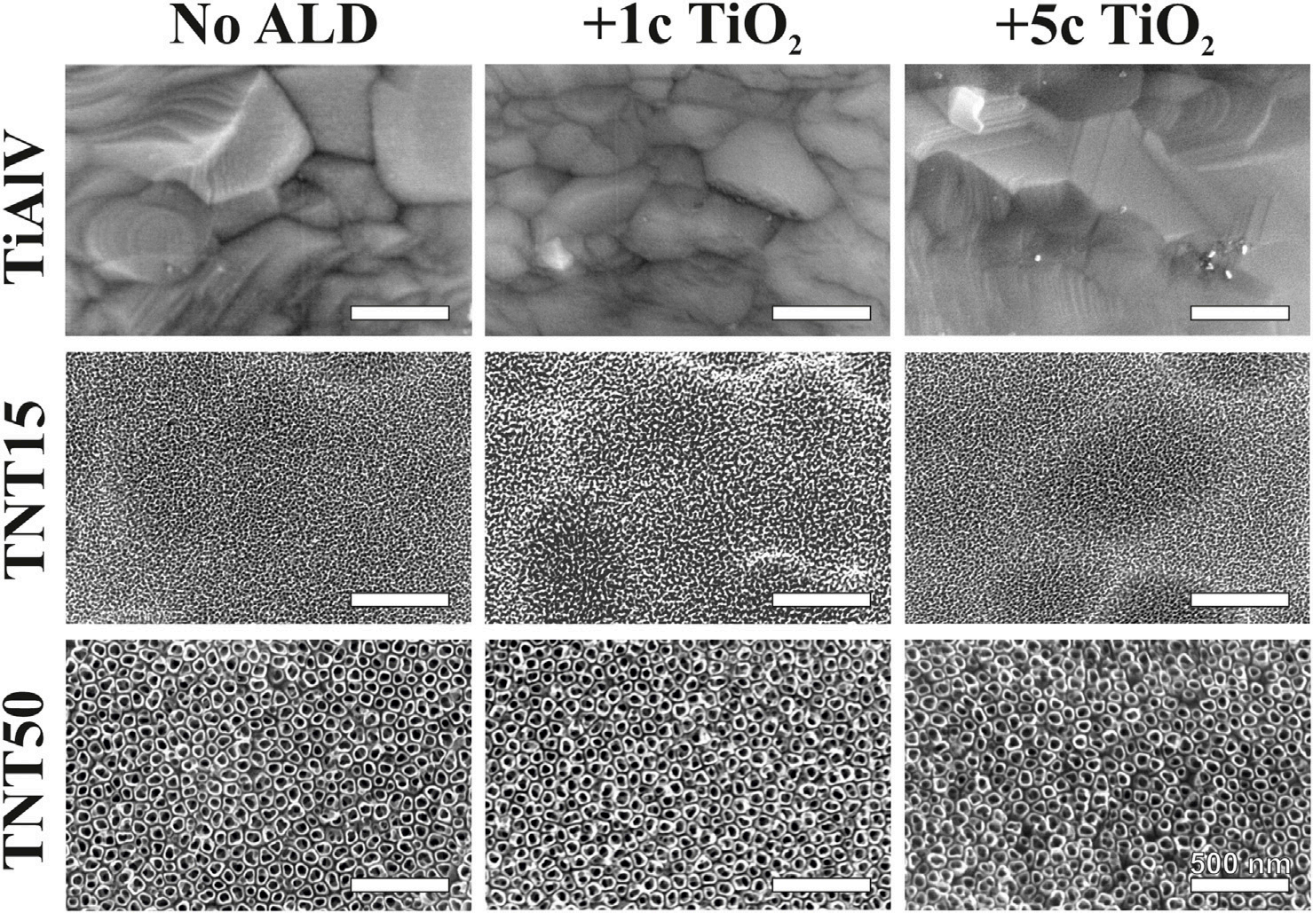
SEM top-view images of TiAlV foils, TNT15, and TNT50 layers before and after 1c and 5c TiO_2_ ALD.

Figure 1 shows the representative top-view SEM images of all TiAlV foils and TNT layers before and after 1c and 5c TiO_2_ ALD coating. Self-organized TNT layers were obtained on TiAlV foils after the anodization. Detailed measurements revealed an inner diameter of ∼15 nm after anodization at 3.3 V (TNT15) and ∼50 nm after anodization at 15 V (TNT50). No significant morphological differences were observed before and after 1c and 5c TiO_2_ ALD coating on TiAlV foils and TNT layers by SEM as the nominal thickness of the 1c and 5c TiO_2_ ALD coating is extremely small (approx. ∼0.055 and ∼0.3 nm, respectively) (Motola et al., 2020).

Figure 1 represents only the anodized α phase of the alloy, while in Supplementary Figure S1 the as-anodized α and β phases are depicted. Nevertheless, α-phase covers approximately 90% of the surface. It is worth noting that the TNT layers reflect the alloy’s two-phase nature. However, as this behavior is consistent across all samples, it can neither be altered, nor controlled, thus it does not raise concerns for our research. The thicknesses of the TNT15 and TNT50 layers before 1c and 5c TiO_2_ ALD coating are shown in Supplementary Figure S2, and the values determined from SEM cross-sectional images were ∼118 nm and ∼410 nm, respectively.

The surface composition in atomic % was assessed through XPS, with the outcomes given in detail in Table 2. Variances in surface atomic composition were observed between uncoated TiAlV foils and those coated with 1c and 5c TiO_2_ ALD, as well as between TNT15 and TNT50 layers. Across all 1c and 5c TiO_2_ ALD-coated TNT layers, the levels of F and N, originating from anodization electrolytes, as well as V and Al, originating from the alloy, decreased. The V was not detectable on the surface of TiAlV foils, which can be explained by its very low content in the native oxide grown on the alloy upon atmospheric influence. In an earlier work (Macak et al., 2005), once sputtering of the alloy surface was conducted, V species could be clearly detected. However, the V was detected as part of the mixed Ti-Al-V oxide on the TNT layers, the amount decreased with the increment of ALD cycles. This can be explained by the fact that TiO_2_ is shading the mentioned elements. On the other hand, C (adventitious carbon) exhibited no significant change after 1c and 5c TiO_2_ ALD coating due to its appearance in the environment.

**TABLE 2:** Surface chemical composition in atomic % before and after 1c TiO_2_, and 5c TiO_2_ ALD coating, on TiAlV foils, TNT15, and TNT50 layers, measured by XPS.

Chemical composition (atomic %)								
Substrate	C	O	N	F	Ti	Al	V	other
TiAlV	28.1	43.89	4.07	-	16.67	4.44	*	2.83
TiAlV + 1c TiO_2_	18.63	47.38	5.21	-	19.66	3.83	*	5.27
TiAlV + 5c TiO_2_	19.64	49.34	3.85	-	19.08	3.23	*	4.87
TNT15	25.13	46.53	1.74	9.34	13.61	2.84	0.71	0.12
TNT15 + 1c TiO_2_	23.63	50.67	1.03	4.39	17.19	2.61	0.48	-
TNT15 + 5c TiO_2_	21.83	52.87	0.95	3.88	18.06	1.96	0.39	0.06
TNT50	31.92	38.46	2.56	15.57	8.46	2.47	0.54	-
TNT50 + 1c TiO_2_	24.65	50.93	1.1	2.81	17.94	2.08	0.49	-
TNT50 + 5c TiO_2_	23.51	53.98	-	2.09	18.41	1.63	0.38	-

* V was not detected due to its too-low content in the native TiO_2_ oxide on the TiAlV foils.

Additionally, the contents of O and Ti increased by adding 1c and 5c TiO_2_ ALD. Simultaneously, the ratio between O and Ti changed after the TiO_2_ ALD coating (i.e., the ratio gets closer to 2:1), which is clear evidence of the high purity of TiO_2_ ALD coatings (Albu et al., 2008; Capek et al., 2024). The reason can be attributed to the uniform TiO_2_ coating, achieved through a robust ALD protocol that ensures sufficient precursors and time for the growth of a TiO_2_ uniform layer (Motola et al., 2020).

### 3.2 Wettability measurements

Table 3 and Supplementary Figure S3 present the WCA values and photographs, respectively, for all materials, before and after 1c and 5c TiO_2_ ALD coatings. As-prepared TNT layers on TiAlV alloy show a hydrophilic behavior due to the formation of hydroxylated TNT layers. This agrees with the results reported by Shin et al. on anodized TiAlV surfaces using F-containing electrolytes (Shin et al., 2011). Nevertheless, after TiO_2_ ALD coating, the TNT layers possess significantly higher contact angles, yielding a somewhat more hydrophobic nature compared to the uncoated TNT layers (Balaur et al., 2005). In fact, according to the literature, the surface wetting properties are influenced by two parameters: surface free energy (SFE) and surface roughness (Neumann and Good, 1972; Giljean et al., 2011). All elements present on the surface of investigated samples exhibit distinct SFE values. Therefore, when WCA measurements are performed on TNT layers formed on TiAlV alloy and modified with TiO_2_ ALD coating, the water’s bonding tendency relies on the SFE of the elements on the surface that contact the water. This can affect the WCA, making the surface area in contact with water smaller, when the elements exhibit a lower SFE (Neumann and Good, 1972; Yuan and Lee, 2013). As a result, the surfaces of TNT15 and TNT50 layers became more hydrophobic after the TiO_2_ ALD coating, with the 5c TiO_2_ ALD-coated TNT15 layer showing the highest WCA values among the TNT layers.

**TABLE 3:** Water contact angles before and after 1c TiO_2_, and 5c TiO_2_ ALD coating on TiAlV foils, TNT15, and TNT50 layers.

Substrate	No ALD	+1c TiO_2_	+5c TiO_2_
TiAlV	74.9° ± 3.5°	71.1° ± 3.2°	69.0° ± 2.4°
TNT15	17.0° ± 0.5°	90.9° ± 2.6°	101.5° ± 2.0°
TNT50	24.8° ± 3.4°	55.5° ± 1.9°	78.2° ± 2.0°

On the other hand, the WCA of non-anodized TiAlV foil surfaces was ∼74.9° due to the low surface free energy present on this alloy consisting of Ti, Al, and V (Neumann and Good, 1972). The deposition of 1c and 5c TiO_2_ ALD coatings on TiAlV foils resulted in slightly enhanced hydrophilic properties of the surfaces, as evidenced by the lower WCA values observed for all foils. In this case, the result is due to the smoothening of the original surface that the TiAlV foils present after the TiO_2_ ALD process. The literature also reported a decrease in the contact angle of TiO_2_ surfaces after the TiO_2_ ALD coating (Liu et al., 2017). Overall, this behavior indicates that the wettability is influenced not only by the surface chemistry (with and without ALD coating) but also by the roughness of surfaces, as shown in the literature (Anselme et al., 2000; Basiaga et al., 2017) and as discussed further.

### 3.3 Surface topography and roughness

The roughness of the TiAlV foils and TNT layers was obtained by AFM measurements. Table 4 shows the root means square (RMS) values representing the roughness deviation calculated for each set of materials. A decrease in the RMS values among TiAlV foils was observed with the increasing TiO_2_ ALD coating thickness. The TiAlV alloy presents a smoother surface compared with the Ti foils presented in our previous studies (Baishya et al., 2023; Capek et al., 2024). This can be related to the different processing techniques, such as rolling, annealing, or other heat treatments, that may be applied to TiAlV and Ti foils, resulting in variations in surface roughness (Wittenauer and Walser, 1990; Wang et al., 2019). On the contrary, the roughness of TNT layers increased after ALD coating in all cases. This increase can be attributed to a combination of surface nucleation effects inherent to the ALD technique (Wind et al., 2009; Puurunen et al., 2011), and mechanical interactions (Zazpe et al., 2016; Baishya et al., 2023). A slight roughness difference among the TNT layers with different diameters can be distinguished and in all cases (whether without or with TiO_2_ ALD coating), the measured roughness was larger for 15 nm TNT layers. These trends are in good correlation with our previous studies on Ti foils (Motola et al., 2020; Capek et al., 2024).

**TABLE 4:** Roughness of TiAlV foils, TNT15, and TNT50 layers before and after 1c TiO_2_, and 5c TiO_2_ ALD coating, respectively.

RMS roughness (nm)			
Substrate	No ALD	+1c TiO_2_	+5c TiO_2_
TiAlV	54.6 ± 17.3	42.8 ± 14.4	39.5 ± 10.5
TNT15	76.1 ± 15.6	82.8 ± 34.5	88.7 ± 32.9
TNT50	64.9 ± 25.0	73.8 ± 9.4	74.8 ± 13.2

The AFM topographical image scans before and after 1c and 5c TiO_2_ ALD coating of TiAlV foils, TNT15, and TNT50 layers are shown in Supplementary Figure S4, S5. One can see that no metallic grains are visible on the surface of TiAlV foils and TNT layers. The remnant grooves, or grain boundaries have disappeared on substrates containing TNT layers after the anodization process (i.e., formation and dissolution of TiO_2_ by the voltage-induced etching of the TiAlV alloy by fluoride ions) (Macak et al., 2005; Motola et al., 2020). Overall, the ALD coating did not detectably change the morphology of all materials, as visualized by the AFM.

### 3.4 Cell growth and proliferation

**FIGURE 2:**
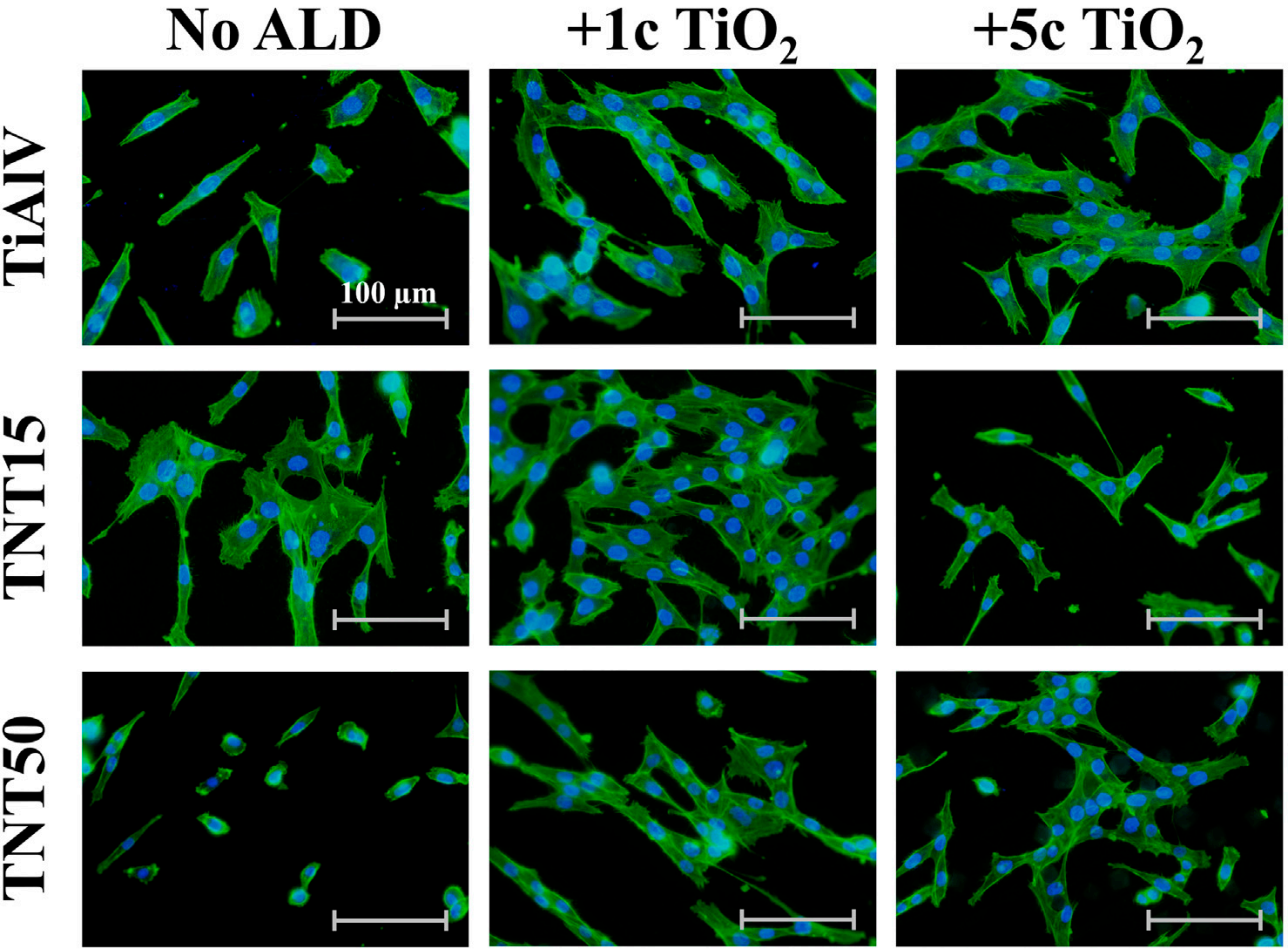
Photomicrographs of MG-63 cells grown on uncoated or 1c and 5c TiO_2_ ALD-coated TiAlV foils, TNT15 and TNT50 layers for 72 h (No ALD = without TiO_2_ ALD coating; 1c ALD = 1c TiO_2_ ALD coating; 5c ALD = 5c TiO_2_ ALD coating). The actin filaments were stained with the Phalloidin-FITC probe (green), and the cell’s nuclei were stained with the Hoechst 33,258 probe (blue).

**FIGURE 3:**
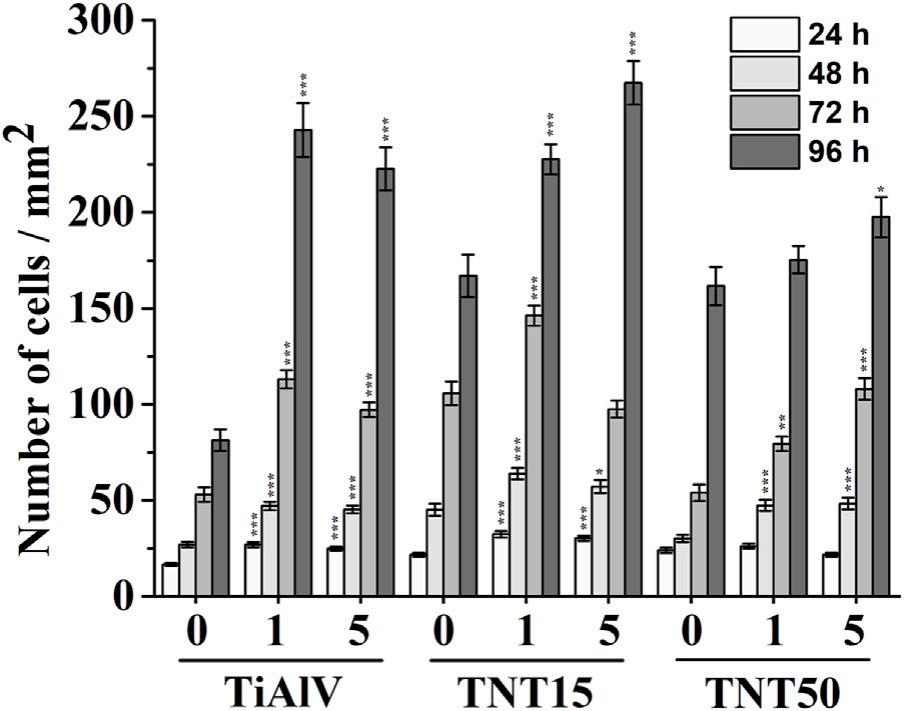
Density of MG-63 cells grown on uncoated or 1c and 5c TiO_2_ ALD-coated TiAlV foils, TNT15 and TNT50 layers for 24–96 h (0 = without TiO_2_ ALD coating; 1 = 1c TiO_2_ ALD coating; 5 = 5c TiO_2_ ALD coating). Data originated from three independent experiments presented as mean ± SEM (*, *p* < 0.05; **, *p* < 0.01; ***, *p* < 0.001, vs 0c at appropriate time interval).

An evaluation of the cell growth, i.e., their adhesion and proliferation, of MG-63 cells during incubation up to 96 h was carried out on TiAlV foils, TNT15, and TNT50 layers with and without 1c and 5c TiO_2_ ALD coating. In the present study, the MG-63 cell line was used, because this type of cell has been frequently used to evaluate cell growth on surfaces modified by the ALD technique (Motola et al., 2020; Nazarov et al., 2022). This study follows our previous works evaluating the biological effects of TiO_2_ ALD coating on nanomaterials (Motola et al., 2020; Capek et al., 2024). The short-term cultivation effect of TiAlV foils, TNT15, and TNT50 layers coated by 1c and 5c TiO_2_ ALD on cell adhesion and proliferation was assessed in this work. Several previous studies evaluated long-term performance in an environment similar to body fluids (Grigal et al., 2012; Abbass et al., 2018). A study by Abbass et al. (2018) proved the corrosion resistance and low toxicity of the material coated by TiO_2_ ALD in long-term incubation (2 weeks) in the simulated body fluid. This is also confirmed by another study (Grigal et al., 2012), where the stability of the amorphous TiO_2_ coating was better compared to the anatase phase. Figure 2 shows photomicrographs of MG-63 cells cultured on TiAlV foils, TNT15, and TNT50 layers, which were uncoated or coated with 1c or 5c TiO_2_ ALD. To identify the functional morphology of MG-63, staining of cell nuclei and actin filaments with fluorescent probes was used according to other authors (Iwata et al., 2017; Li et al., 2020). Subsequently, the images were subjected to image analysis. Cell nuclei were counted in individual fields of view and related to an area (=mm^2^), which is also a common approach for the quantification of cell growth in other studies (Kim et al., 2005; Zhang et al., 2012). Figure 3 shows an increased number of cells in all 1c and 5c TiO_2_ ALD-coated materials (TiAlV foils, TNT15, and TNT50 layers), compared to their uncoated counterparts at all time intervals. The highest increases in cell densities were observed in both TiAlV foils and TNT15 layers with 1c and 5c TiO_2_ ALD coating, compared to the uncoated ones after 96 h. As the doubling time of the MG-63 cells was 31 h, it can be assumed that the TiO_2_ coating had a beneficial effect not only on the cell adhesion but also on the cell proliferation. Other reports used a wide range of TiO_2_ ALD film thicknesses achieved by a range of ALD cycles, i.e., 1-250c (Motola et al., 2020; Capek et al., 2024), 250-1000c (Huang et al., 2019), and more than 1000c TiO_2_ (Huang et al., 2019).

WCA measurements shown in Table 3 provided findings of decreased hydrophilicity for all coated TNTs (i.e., 1c and 5c TiO_2_ ALD) compared to uncoated ones. In the case of the TiAlV foil, the wettability remained more or less unchanged after TiO_2_ ALD coatings. To ensure the cell adhesion to a surface, the optimal range of water contact angles has been reported in several studies to be between 60° and 80° (Arima and Iwata, 2007; Kim et al., 2007; 2015; Lv et al., 2015; Yao et al., 2019). Our results showed that WCA in both tested TNTs after 1c and 5c TiO_2_ ALD reached values nearer to the optimal range of 60°–80° in comparison to WCA values of uncoated TNTs. However, WCA is not the only factor influencing cell growth, the whole situation is more complex. The number of MG-63 cells cultured on uncoated TiAlV foils (as such or with TNT layers) was lower compared to coated counterparts with 1c and 5c TiO_2_ ALD, as shown in Figures 2, 3. In particular, the increased cell number can be assigned to the reduction of the cytotoxic effect of V and F elements that are present on the uncoated surfaces. In samples coated with 1c and 5c TiO_2_ ALD, the concentrations of V and F elements were reduced (as demonstrated by XPS results in Table 2) due to the shading effect that TiO_2_ ALD coating gives to the surfaces and thus their cytotoxic effect was reduced. This effect was already described in the literature, either as the negative effect of V and F elements on the cell proliferation (Goc, 2006; Szewczenko et al., 2010), or the possible involvement of V and its compounds in the induction of the formation of ROS, which can suppress cell growth (Cortizo et al., 2000; Aureliano et al., 2023).

### 3.5 Cell elongation

**FIGURE 4:**
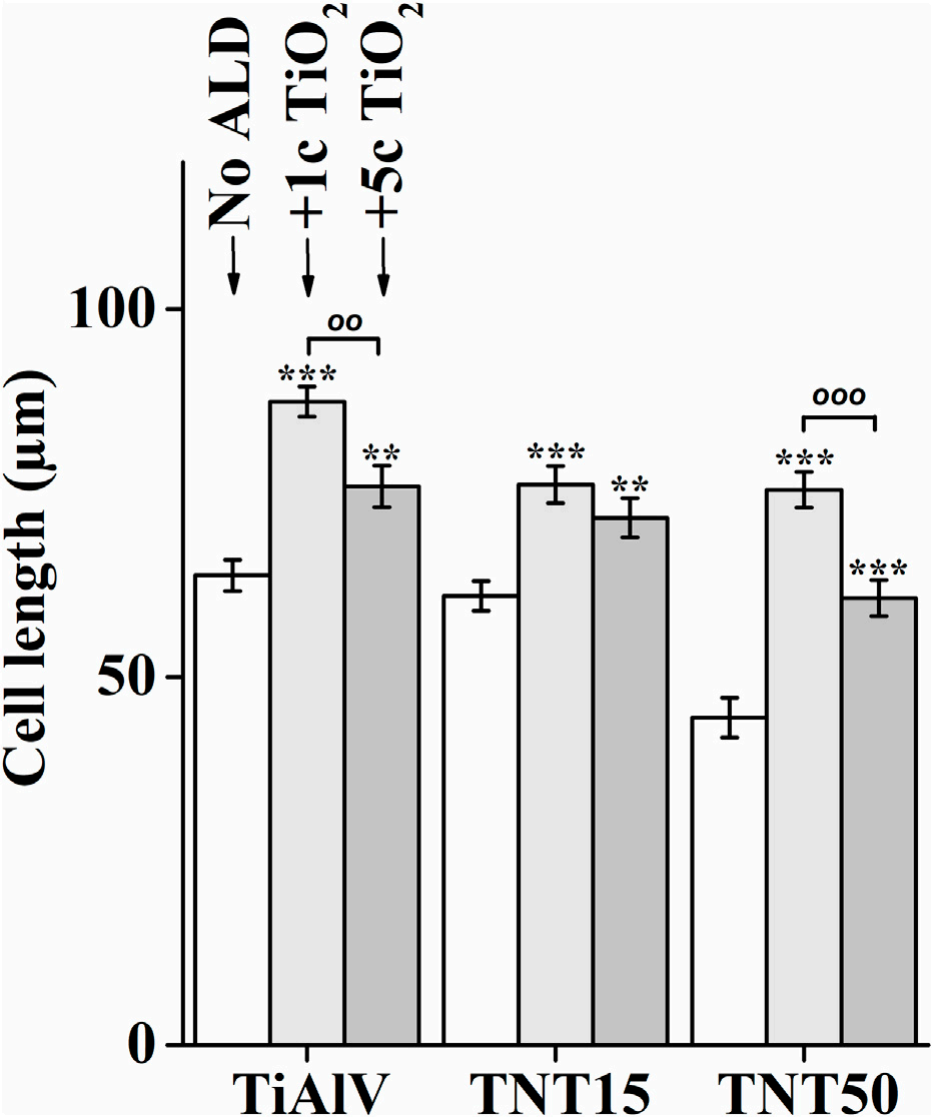
Analysis of elongation of MG-63 cells grown on uncoated or 1c and 5c TiO_2_ ALD-coated TiAlV foils, TNT15 and TNT50 layers for 72 h (No ALD = without TiO_2_ ALD coating; 1c ALD = 1c TiO_2_ ALD coating; 5c ALD = 5c TiO_2_ ALD coating). Data originated from three independent experiments presented as mean ± SEM (**, *p* < 0.01; ***, *p* < 0.001, vs 0c; ^*oo*^, *p* < 0.01; ^*ooo*^, *p* < 0.001, 1c vs 5c).

The cell elongation was evaluated from photomicrographs obtained (examples of which are given in Figure 2), as it correlates with increased adhesion of cells to a material (Motola et al., 2020; Nazarov et al., 2022). Figure 4 shows the resulting analysis of the elongation of MG-63 cells incubated for 72 h on TiAlV foils, TNT15, and TNT50 layers with and without 1c and 5c TiO_2_ ALD coating. Cells cultured in TiAlV foils and TNT layers with TiO_2_ ALD coatings had a more elongated structure, compared to those cultured in uncoated samples. An increase in elongation by approximately 20% was observed in cells cultured on TiAlV foils, TNT15, and TNT50 layers coated with 1c and 5c TiO_2_ ALD compared to uncoated samples. Interestingly, cells grown on TiAlV foils and TNT50 layers coated with 1c TiO_2_ ALD had a significantly increased elongated structure compared to materials coated with 5c TiO_2_ ALD. The increase in the elongation in cells on TiO_2_ ALD coated materials compared to uncoated materials can be attributed to the achievement of WCA values in the optimal range of 60°–80°. The difference in the elongation of cells grown on 1c and 5c TiO_2_ ALD coated materials could be likely ascribed to the combined effects of differing wettability and surface structure and chemistry, according to AFM and XPS results. The photomicrographs of MG-63 cells after 24, 48, and 96 h are provided as supplementary data in Supplementary Figures S6–S8, and the evaluation of elongation in MG-63 cells grown on TiAlV foils, TNT15, and TNT50 layers for 24 and 48 h is provided as supplementary data in Supplementary Figure S9. MG-63 cell elongation analysis was not performed in the 96-h time interval due to the high cell confluence that is nearly 100%.

The increased number of elongated cells after cultivation on materials modified by TiO_2_ ALD was also observed in several studies by other authors (Chuang et al., 2021; Abushahba et al., 2023). For instance, the elongated structure of human dental pulp stem cells was observed after culture on Si substrates coated with 50c TiO_2_ after 24 h. The cells grown on ALD-modified Si substrates exhibited higher average aspect ratios (i.e., ratio of the cellular major and minor axes) compared to unmodified materials (Chuang et al., 2021). The MC3T3-E1 osteoblasts growth on Ti sheets with ALD coating have been studied in the literature (Kylmäoja et al., 2022; Abushahba et al., 2023), showing an elongated structure for instance with/without 4000 cycles of hydroxyapatite coating were assessed after 48 h (Kylmäoja et al., 2022). Cells on hydroxyapatite-coated Ti sheets had higher average aspect ratios compared to uncoated Ti sheets. For cells grown on Ti sheets, a circular structure was detected in the contrast to cells grown on Ti sheets coated with hydroxyapatite having elongated morphology.

## 4 Conclusion

The influence of the surface morphology and chemical composition of TiAlV foils and TNT layers grown on TiAlV foils on the cell growth of MG-63 osteoblastic cells before and after 1c or 5c TiO_2_ ALD coating was investigated for the first time. The addition of ultrathin TiO_2_ ALD coatings improved the biocompatibility of TNT layers for the cell growth. All the surfaces were evaluated in terms of morphology, composition, and wettability. SEM images did not show any significant changes in the morphology of surfaces after 1c and 5c TiO_2_ ALD coating in any of the substrates. However, XPS analysis revealed that the chemical composition of all surfaces changed after 1c and 5c TiO_2_ ALD coating, resulting in a decrease in the occurrence of F and V elements. WCA results demonstrated that the surface remained stably hydrophilic for TiAlV foils after 1c and 5c TiO_2_ ALD coating, contrary to the case of TNT layers, when the WCA strongly increased after 1c and 5c TiO_2_ ALD coating towards more hydrophobic character. The change of WCA was probably caused by changes in the surface free energy and the roughness after TiO_2_ ALD coatings. The AFM results showed a significant increase in the roughness after TiO_2_ ALD coating on all TNT layers, but for the TiAlV foils the roughness did not change very significantly. In terms of the biocompatibility, the growth of MG-63 cells on all materials was estimated. The cell growth evaluation showed a significant increase in the number of cells cultivated on TiAlV foils, TNT15 or TNT50 layers coated with 1c or 5c TiO_2_ ALD, in comparison to uncoated samples. These outcomes were supported by findings of elongated cells cultured on all tested 1c or 5c TiO_2_ ALD coated materials demonstrating more beneficial circumstances for cell growth after 1c or 5c TiO_2_ ALD coating. This effect is ensured by the combination of F and V shading and achievement of WCA values near the range of 60°–80° being optimal for the cell adhesion to a surface in general. Overall, the results presented here bring new and very valuable findings, on how to increase the biocompatibility of TiAlV-derived materials using ultra-thin TiO_2_ ALD coating, although additional detailed cellular studies are required to describe the exact mechanism accounting for the increased proliferation of MG63 cells.

## Data Availability

The original contributions presented in the study are included in the article/Supplementary Material, further inquiries can be directed to the corresponding authors.

## References

[B1] Abbass M. K. Ajeel S. A. Wadullah H. M. (2018). “Biocompatibility, bioactivity and corrosion resistance of stainless steel 316L nanocoated with TiO2and Al2O3by atomic layer deposition method,” in Journal of physics: conference series (Bristol, United Kingdom: IOP Publishing), 012017.

[B2] Abushahba F. Areid N. Kylmäoja E. Holopainen J. Ritala M. Hupa L. (2023). Effect of atomic-layer-deposited hydroxyapatite coating on surface thrombogenicity of titanium. Coatings 13, 1810. 10.3390/coatings13101810

[B3] Albu S. P. Ghicov A. Aldabergenova S. Drechsel P. LeClere D. Thompson G. E. (2008). Formation of double-walled TiO2 nanotubes and robust anatase membranes. Adv. Mater. 20, 4135–4139. 10.1002/adma.200801189

[B4] Anselme K. Linez P. Bigerelle M. Le Maguer D. Le Maguer A. Hardouin P. (2000). The relative influence of the topography and chemistry of TiAl6V4 surfaces on osteoblastic cell behaviour. Biomaterials 21, 1567–1577. 10.1016/s0142-9612(00)00042-9 10885729

[B5] Arima Y. Iwata H. (2007). Effect of wettability and surface functional groups on protein adsorption and cell adhesion using well-defined mixed self-assembled monolayers. Biomaterials 28, 3074–3082. 10.1016/j.biomaterials.2007.03.013 17428532

[B6] Aureliano M. De Sousa-Coelho A. L. Dolan C. C. Roess D. A. Crans D. C. (2023). Biological consequences of vanadium effects on formation of reactive oxygen species and lipid peroxidation. Int. J. Mol. Sci. 24, 5382. 10.3390/ijms24065382 36982458 PMC10049017

[B7] Baishya K. Vrchovecká K. Alijani M. Rodriguez-Pereira J. Thalluri S. M. Goldbergová M. P. (2023). Bio-AFM exploits enhanced response of human gingival fibroblasts on TiO2 nanotubular substrates with thin TiO2 coatings. Appl. Surf. Sci. Adv. 18, 100459. 10.1016/j.apsadv.2023.100459

[B8] Balaur E. Macak J. M. Taveira L. Schmuki P. (2005). Tailoring the wettability of TiO2 nanotube layers. Electrochem Commun. 7, 1066–1070. 10.1016/j.elecom.2005.07.014

[B9] Basiaga M. Walke W. Staszuk M. Kajzer W. Kajzer A. Nowińska K. (2017). Influence of ALD process parameters on the physical and chemical properties of the surface of vascular stents. Archives Civ. Mech. Eng. 17, 32–42. 10.1016/j.acme.2016.08.001

[B10] Campoccia D. Montanaro L. Arciola C. R. (2006). The significance of infection related to orthopedic devices and issues of antibiotic resistance. Biomaterials 27, 2331–2339. 10.1016/j.biomaterials.2005.11.044 16364434

[B11] Capek J. Sepúlveda M. Bacova J. Rodriguez-Pereira J. Zazpe R. Cicmancova V. (2024). Ultrathin TiO2 coatings via atomic layer deposition strongly improve cellular interactions on planar and nanotubular biomedical Ti substrates. ACS Appl. Mater Interfaces 16, 5627–5636. 10.1021/acsami.3c17074 38275195 PMC10859894

[B12] Chen Q. Thouas G. A. (2015). Metallic implant biomaterials. Mater. Sci. Eng. R Rep. 87, 1–57. 10.1016/j.mser.2014.10.001

[B13] Chuang Y.-C. Wang L. Feng K.-C. Subramanian A. Chang C.-C. Simon M. (2021). The role of titania surface coating by atomic layer deposition in improving osteogenic differentiation and hard tissue formation of dental pulp stem cells. Adv. Eng. Mater 23, 2100097. 10.1002/adem.202100097

[B14] Cortizo A. M. Bruzzone L. Molinuevo S. Etcheverry S. B. (2000). A possible role of oxidative stress in the vanadium-induced cytotoxicity in the MC3T3E1 osteoblast and UMR106 osteosarcoma cell lines. Toxicology 147, 89–99. 10.1016/s0300-483x(00)00181-5 10874156

[B15] Costa B. C. Tokuhara C. K. Rocha L. A. Oliveira R. C. Lisboa-Filho P. N. Pessoa J. C. (2019). Vanadium ionic species from degradation of Ti-6Al-4V metallic implants: *in vitro* cytotoxicity and speciation evaluation. Mater. Sci. Eng. C 96, 730–739. 10.1016/j.msec.2018.11.090 30606586

[B16] Darwish G. Huang S. Knoernschild K. Sukotjo C. Campbell S. Bishal A. K. (2019). Improving polymethyl methacrylate resin using a novel titanium dioxide coating. J. Prosthodont. 28, 1011–1017. 10.1111/jopr.13032 30720223

[B17] De Maeztu M. A. Alava J. I. Gay-Escoda C. (2003). Ion implantation: surface treatment for improving the bone integration of titanium and Ti6Al4V dental implants. Clin. Oral Implants Res. 14, 57–62. 10.1034/j.1600-0501.2003.140108.x 12562366

[B18] Filova E. Fojt J. Kryslova M. Moravec H. Joska L. Bacakova L. (2015). The diameter of nanotubes formed on Ti-6Al-4V alloy controls the adhesion and differentiation of Saos-2 cells. Int. J. Nanomedicine 10, 7145–7163. 10.2147/ijn.s87474 26648719 PMC4664495

[B19] Giljean S. Bigerelle M. Anselme K. Haidara H. (2011). New insights on contact angle/roughness dependence on high surface energy materials. Appl. Surf. Sci. 257, 9631–9638. 10.1016/j.apsusc.2011.06.088

[B20] Gittens R. A. Olivares-Navarrete R. Schwartz Z. Boyan B. D. (2014). Implant osseointegration and the role of microroughness and nanostructures: lessons for spine implants. Acta Biomater. 10, 3363–3371. 10.1016/j.actbio.2014.03.037 24721613 PMC4103432

[B21] Goc A. (2006). Biological activity of vanadium compounds. Cent. Eur. J. Biol. 1, 314–332. 10.2478/s11535-006-0029-z

[B22] Gomes C. C. Moreira L. M. Santos V. J. S. V. Ramos A. S. Lyon J. P. Soares C. P. (2011). Assessment of the genetic risks of a metallic alloy used in medical implants. Genet. Mol. Biol. 34, 116–121. 10.1590/s1415-47572010005000118 21637553 PMC3085356

[B23] González A. S. Riego A. Vega V. García J. Galié S. Gutierrez del Rio I. (2021). Functional antimicrobial surface coatings deposited onto nanostructured 316L food-grade stainless steel. Nanomaterials 11, 1055. 10.3390/nano11041055 33924070 PMC8074267

[B24] Grigal I. P. Markeev A. M. Gudkova S. A. Chernikova A. G. Mityaev A. S. Alekhin A. P. (2012). Correlation between bioactivity and structural properties of titanium dioxide coatings grown by atomic layer deposition. Appl. Surf. Sci. 258, 3415–3419. 10.1016/j.apsusc.2011.11.082

[B25] Huang L. Su K. Zheng Y.-F. Yeung K. W.-K. Liu X.-M. (2019). Construction of TiO2/silane nanofilm on AZ31 magnesium alloy for controlled degradability and enhanced biocompatibility. Rare Met. 38, 588–600. 10.1007/s12598-018-1187-7

[B26] Im C. Park J.-H. Jeon Y.-M. Kim J.-G. Jang Y.-S. Lee M.-H. (2022). Improvement of osseointegration of Ti–6Al–4V ELI alloy orthodontic mini-screws through anodization, cyclic pre-calcification, and heat treatments. Prog. Orthod. 23, 11. 10.1186/s40510-022-00405-8 35368222 PMC8977256

[B27] Iwata N. Nozaki K. Horiuchi N. Yamashita K. Tsutsumi Y. Miura H. (2017). Effects of controlled micro-/nanosurfaces on osteoblast proliferation. J. Biomed. Mater Res. A 105, 2589–2596. 10.1002/jbm.a.36118 28544516

[B28] Kim H. J. Kim S. H. Kim M. S. Lee E. J. Oh H. G. Oh W. M. (2005). Varying Ti-6Al-4V surface roughness induces different early morphologic and molecular responses in MG63 osteoblast-like cells. J. Biomed. Mater. Res. Part A Official J. Soc. Biomaterials, Jpn. Soc. Biomaterials, Aust. Soc. Biomaterials Korean Soc. Biomaterials 74, 366–373. 10.1002/jbm.a.30327 15983984

[B29] Kim S. H. Ha H. J. Ko Y. K. Yoon S. J. Rhee J. M. Kim M. S. (2007). Correlation of proliferation, morphology and biological responses of fibroblasts on LDPE with different surface wettability. J. Biomater. Sci. Polym. Ed. 18, 609–622. 10.1163/156856207780852514 17550662

[B30] Kim T. Sridharan I. Zhu B. Orgel J. Wang R. (2015). Effect of CNT on collagen fiber structure, stiffness assembly kinetics and stem cell differentiation. Mater. Sci. Eng. C 49, 281–289. 10.1016/j.msec.2015.01.014 PMC7225775 25686951

[B31] Klokkevold P. R. Nishimura R. D. Adachi M. Caputo A. (1997). Osseointegration enhanced by chemical etching of the titanium surface. A torque removal study in the rabbit. Clin. Oral Implants Res. 8, 442–447. 10.1034/j.1600-0501.1997.080601.x 9555202

[B32] Konttinen Y. T. Pajarinen J. (2013). Adverse reactions to metal-on-metal implants. Nat. Rev. Rheumatol. 9, 5–6. 10.1038/nrrheum.2012.218 23208186

[B33] Korbecki J. Baranowska-Bosiacka I. Gutowska I. Chlubek D. (2015). Vanadium compounds as pro-inflammatory agents: effects on cyclooxygenases. Int. J. Mol. Sci. 16, 12648–12668. 10.3390/ijms160612648 26053397 PMC4490466

[B34] Kylmäoja E. Holopainen J. Abushahba F. Ritala M. Tuukkanen J. (2022). Osteoblast attachment on titanium coated with hydroxyapatite by atomic layer deposition. Biomolecules 12, 654. 10.3390/biom12050654 35625580 PMC9138598

[B35] Lee K. Mazare A. Schmuki P. (2014). One-dimensional titanium dioxide nanomaterials: nanotubes. Chem. Rev. 114, 9385–9454. 10.1021/cr500061m 25121734

[B36] Li Y. Wang S. Dong Y. Mu P. Yang Y. Liu X. (2020). Effect of size and crystalline phase of TiO2 nanotubes on cell behaviors: a high throughput study using gradient TiO2 nanotubes. Bioact. Mater 5, 1062–1070. 10.1016/j.bioactmat.2020.07.005 32695936 PMC7363987

[B37] Liu L. Bhatia R. Webster T. J. (2017). Atomic layer deposition of nano-TiO2 thin films with enhanced biocompatibility and antimicrobial activity for orthopedic implants. Int. J. Nanomedicine 12, 8711–8723. 10.2147/ijn.s148065 29263665 PMC5724422

[B38] Long M. Rack H. J. (1998). Titanium alloys in total joint replacement—a materials science perspective. Biomaterials 19, 1621–1639. 10.1016/s0142-9612(97)00146-4 9839998

[B39] Luo B. Yang H. Liu S. Fu W. Sun P. Yuan M. (2008). Fabrication and characterization of self-organized mixed oxide nanotube arrays by electrochemical anodization of Ti–6Al–4V alloy. Mater Lett. 62, 4512–4515. 10.1016/j.matlet.2008.08.015

[B40] Lv L. Liu Y. Zhang P. Zhang X. Liu J. Chen T. (2015). The nanoscale geometry of TiO2 nanotubes influences the osteogenic differentiation of human adipose-derived stem cells by modulating H3K4 trimethylation. Biomaterials 39, 193–205. 10.1016/j.biomaterials.2014.11.002 25468371

[B41] Macak J. M. Hildebrand H. Marten-Jahns U. Schmuki P. (2008). Mechanistic aspects and growth of large diameter self-organized TiO2 nanotubes. J. Electroanal. Chem. 621, 254–266. 10.1016/j.jelechem.2008.01.005

[B42] Macak J. M. Schmuki P. (2006). Anodic growth of self-organized anodic TiO2 nanotubes in viscous electrolytes. Electrochim Acta 52, 1258–1264. 10.1016/j.electacta.2006.07.021

[B43] Macak J. M. Tsuchiya H. Taveira L. Ghicov A. Schmuki P. (2005). Self-organized nanotubular oxide layers on Ti-6Al-7Nb and Ti-6Al-4V formed by anodization in NH4F solutions. J. Biomed. Mater Res. A 75, 928–933. 10.1002/jbm.a.30501 16138327

[B44] Magesh S. Vasanth G. Revathi A. Geetha M. (2018). “Use of nanostructured materials in implants,” in Nanobiomaterials (Elsevier), 481–501.

[B45] Matykina E. Conde A. De Damborenea J. y Marero D. M. Arenas M. A. (2011). Growth of TiO2-based nanotubes on Ti–6Al–4V alloy. Electrochim Acta 56, 9209–9218. 10.1016/j.electacta.2011.07.131

[B46] Matykina E. Monfort F. Berkani A. Skeldon P. Thompson G. E. Gough J. (2007). Characterization of spark-anodized titanium for biomedical applications. J. Electrochem Soc. 154, C279. 10.1149/1.2717383

[B47] Motola M. Capek J. Zazpe R. Bacova J. Hromadko L. Bruckova L. (2020). Thin TiO2 coatings by ALD enhance the cell growth on TiO2 nanotubular and flat substrates. ACS Appl. Bio Mater 3, 6447–6456. 10.1021/acsabm.0c00871 35021776

[B48] Nazarov D. Ezhov I. Yudintceva N. Shevtsov M. Rudakova A. Kalganov V. (2022). Antibacterial and osteogenic properties of Ag nanoparticles and Ag/TiO2 nanostructures prepared by atomic layer deposition. J. Funct. Biomater. 13, 62. 10.3390/jfb13020062 35645270 PMC9149969

[B49] Neumann A. W. Good R. J. (1972). Thermodynamics of contact angles. I. Heterogeneous solid surfaces. J. Colloid Interface Sci. 38, 341–358. 10.1016/0021-9797(72)90251-2

[B50] Nune K. C. Misra R. D. K. Gai X. Li S. J. Hao Y. L. (2018). Surface nanotopography-induced favorable modulation of bioactivity and osteoconductive potential of anodized 3D printed Ti-6Al-4V alloy mesh structure. J. Biomater. Appl. 32, 1032–1048. 10.1177/0885328217748860 29249195

[B51] Oliveira N. T. C. Ferreira E. A. Duarte L. T. Biaggio S. R. Rocha-Filho R. C. Bocchi N. (2006). Corrosion resistance of anodic oxides on the Ti–50Zr and Ti–13Nb–13Zr alloys. Electrochim Acta 51, 2068–2075. 10.1016/j.electacta.2005.07.015

[B52] Park J. Bauer S. Schlegel K. A. Neukam F. W. von der Mark K. Schmuki P. (2009). TiO2 nanotube surfaces: 15 nm—an optimal length scale of surface topography for cell adhesion and differentiation. small 5, 666–671. 10.1002/smll.200801476 19235196

[B53] Pessoa R. S. Dos Santos V. P. Cardoso S. B. Doria A. Figueira F. R. Rodrigues B. V. M. (2017). TiO2 coatings via atomic layer deposition on polyurethane and polydimethylsiloxane substrates: properties and effects on C. albicans growth and inactivation process. Appl. Surf. Sci. 422, 73–84. 10.1016/j.apsusc.2017.05.254

[B54] Puurunen R. L. Sajavaara T. Santala E. Miikkulainen V. Saukkonen T. Laitinen M. (2011). Controlling the crystallinity and roughness of atomic layer deposited titanium dioxide films. J. Nanosci. Nanotechnol. 11, 8101–8107. 10.1166/jnn.2011.5060 22097537

[B55] Roy P. Berger S. Schmuki P. (2011). TiO2 nanotubes: synthesis and applications. Angew. Chem. Int. Ed. 50, 2904–2939. 10.1002/anie.201001374 21394857

[B56] Sarraf M. Rezvani Ghomi E. Alipour S. Ramakrishna S. Liana Sukiman N. (2021). A state-of-the-art review of the fabrication and characteristics of titanium and its alloys for biomedical applications. Biodes Manuf. 5, 371–395. 10.1007/s42242-021-00170-3 34721937 PMC8546395

[B57] Shin D. H. Shokuhfar T. Choi C. K. Lee S.-H. Friedrich C. (2011). Wettability changes of TiO2 nanotube surfaces. Nanotechnology 22, 315704. 10.1088/0957-4484/22/31/315704 21727317

[B58] Singh R. Dahotre N. B. (2007). Corrosion degradation and prevention by surface modification of biometallic materials. J. Mater Sci. Mater Med. 18, 725–751. 10.1007/s10856-006-0016-y 17143737

[B59] Subramani K. Lavenus S. Rozé J. Louarn G. Layrolle P. (2018) “Impact of nanotechnology on dental implants,” in Emerging nanotechnologies in dentistry, 83–97.

[B60] Szewczenko J. Walke W. Nowinska K. Marciniak J. (2010). Corrosion resistance of Ti-6Al-4V alloy after diverse surface treatments. Materwiss Werksttech 41, 360–371. 10.1002/mawe.201000610

[B61] Tsuchiya H. Berger S. Macak J. M. Ghicov A. Schmuki P. (2007). Self-organized porous and tubular oxide layers on TiAl alloys. Electrochem Commun. 9, 2397–2402. 10.1016/j.elecom.2007.07.013

[B62] Vaithilingam J. Prina E. Goodridge R. D. Hague R. J. M. Edmondson S. Rose F. R. A. J. (2016). Surface chemistry of Ti6Al4V components fabricated using selective laser melting for biomedical applications. Mater. Sci. Eng. C 67, 294–303. 10.1016/j.msec.2016.05.054 27287125

[B63] Vasconcelos D. M. Santos S. G. Lamghari M. Barbosa M. A. (2016). The two faces of metal ions: from implants rejection to tissue repair/regeneration. Biomaterials 84, 262–275. 10.1016/j.biomaterials.2016.01.046 26851391

[B64] Wang L. Xie L. Shen P. Fan Q. Wang W. Wang K. (2019). Surface microstructure and mechanical properties of Ti-6Al-4V/Ag nanocomposite prepared by FSP. Mater Charact. 153, 175–183. 10.1016/j.matchar.2019.05.002

[B65] Wind R. W. Fabreguette F. H. Sechrist Z. A. George S. M. (2009). Nucleation period, surface roughness, and oscillations in mass gain per cycle during W atomic layer deposition on Al2O3. J. Appl. Phys. 105. 10.1063/1.3103254

[B66] Wittenauer J. Walser B. (1990). Processing and properties of titanium foils. Mater. Sci. Eng. A 123, 45–52. 10.1016/0921-5093(90)90208-k

[B67] Yao L. Wu X. Wu S. Pan X. Tu J. Chen M. (2019). Atomic layer deposition of zinc oxide on microrough zirconia to enhance osteogenesis and antibiosis. Ceram. Int. 45, 24757–24767. 10.1016/j.ceramint.2019.08.216

[B68] Yuan Y. Lee T. R. (2013). “Contact angle and wetting properties,” in Surface science techniques (Springer), 3–34.

[B69] Zazpe R. Knaut M. Sopha H. Hromadko L. Albert M. Prikryl J. (2016). Atomic layer deposition for coating of high aspect ratio TiO2 nanotube layers. Langmuir 32, 10551–10558. 10.1021/acs.langmuir.6b03119 27643411 PMC5072108

[B70] Zhang W. Li Z. Liu Y. Ye D. Li J. Xu L. (2012). Biofunctionalization of a titanium surface with a nano-sawtooth structure regulates the behavior of rat bone marrow mesenchymal stem cells. Int. J. Nanomedicine 7, 4459–4472. 10.2147/ijn.s33575 22927760 PMC3422101

[B71] Zhao J. Castranova V. (2011). Toxicology of nanomaterials used in nanomedicine. J. Toxicol. Environ. Health, Part B 14, 593–632. 10.1080/10937404.2011.615113 22008094

