# Supplementary material for: Enhancement of biocompatibility of anodic nanotube structures on biomedical Ti–6Al–4V alloy via ultrathin TiO2 coatings
Source: Front Bioeng Biotechnol. 2024 Dec 2;12:1515810. doi: 10.3389/fbioe.2024.1515810 (PMC11646768; doi:10.3389/fbioe.2024.1515810)
Supplement: Supplementary file 1 [file DataSheet1.docx]

Supplementary Material

**Enhancement of biocompatibility of anodic nanotube structures on biomedical Ti–6Al–4V alloy via ultrathin TiO_2_ coatings**

**Marcela Sepúlveda^1,2^, Jan Capek^3^, Kaushik Baishya^2^, Jhonatan Rodriguez-Pereira^1,2^, Jana Bacova^3^, Stepanka Jelinkova^3^, Raul Zazpe^1,2^, Hanna Sopha^1,2^, Tomas Rousar^3*^ and Jan M. Macak^1,2*^**

^1^ Center of Materials and Nanotechnologies, Faculty of Chemical Technology, University of Pardubice, Nam. Cs. Legii 565, 530 02 Pardubice, Czech Republic

^2^ Central European Institute of Technology, Brno University of Technology, Purkyňova 123, 61200 Brno, Czech Republic

^3^ Department of Biological and Biochemical Sciences, Faculty of Chemical Technology, University of Pardubice, Studentska 573, 532 10 Pardubice, Czech Republic


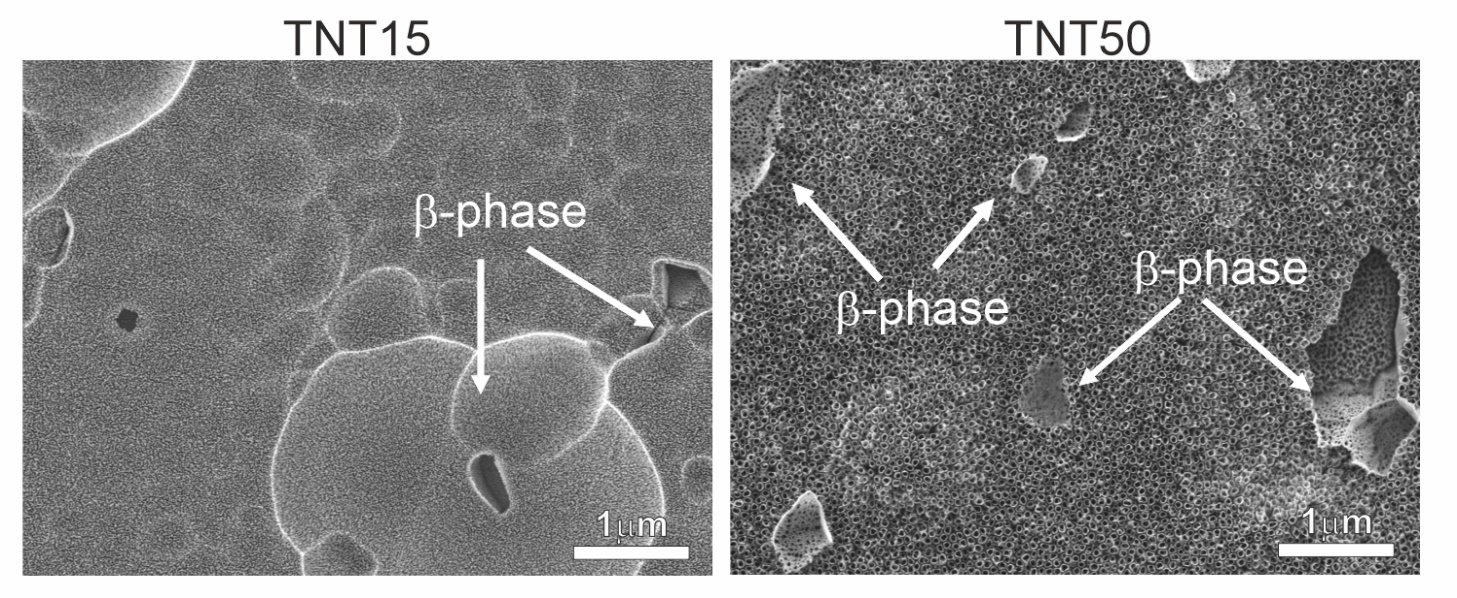


Figure S1. SEM top-view of the TNT layers before TiO_2_ ALD coating showing α + β phases of the anodized TiAlV surface.


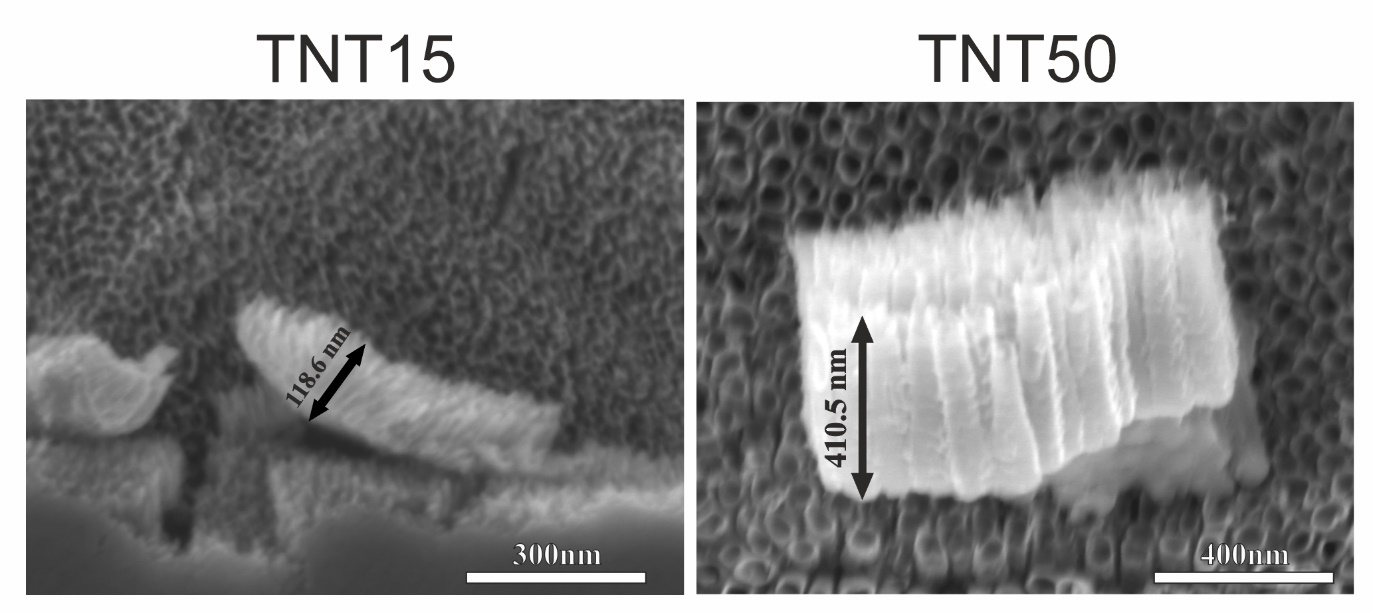


**Figure S2.** SEM cross-sectional views of the TNT layers before TiO_2_ ALD coatings.


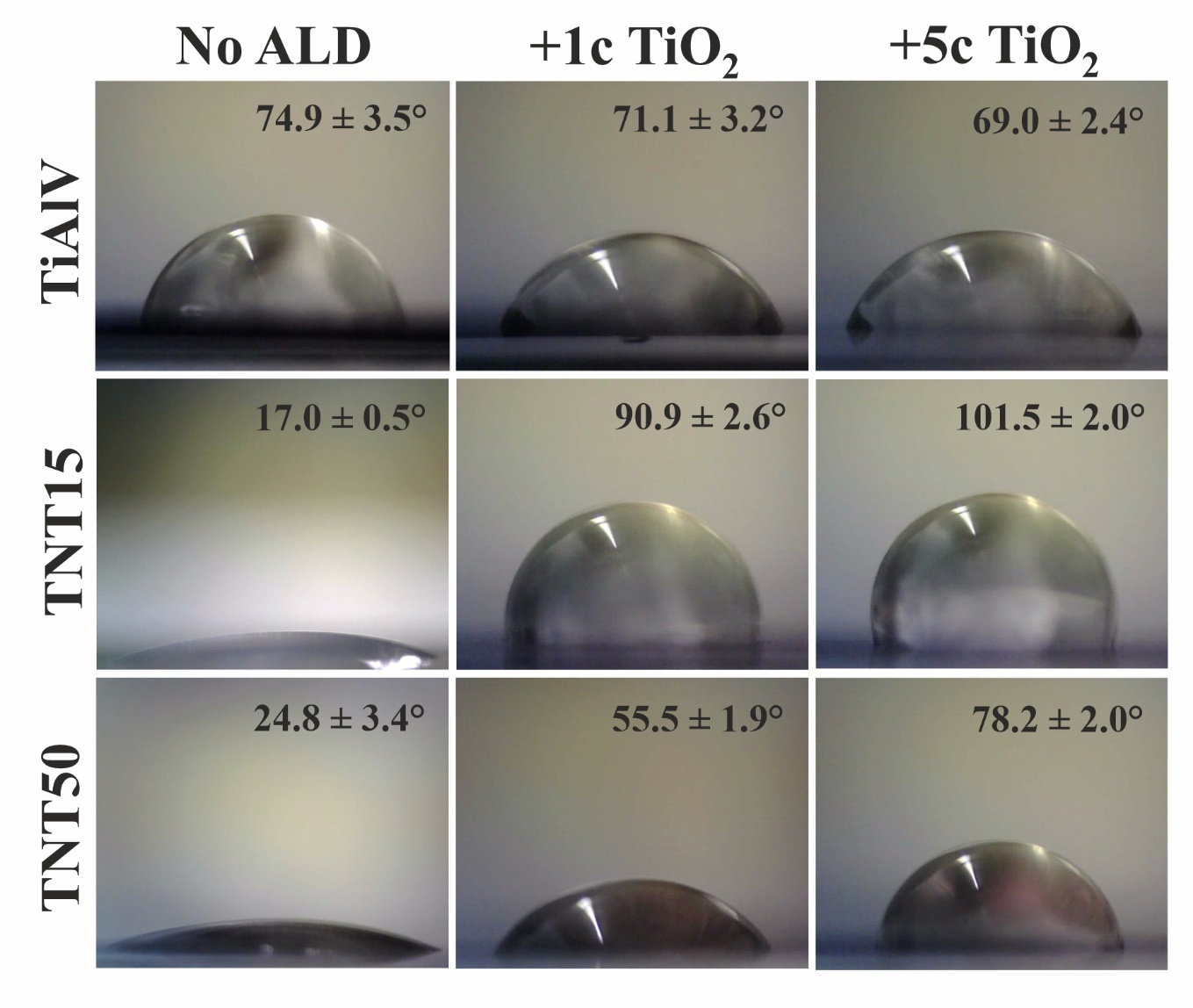


**Figure S3.** Contact angle values and water droplets before and after 1c and 5c TiO_2_ ALD coating on TiAlV foils, TNT15, and TNT50 layers.


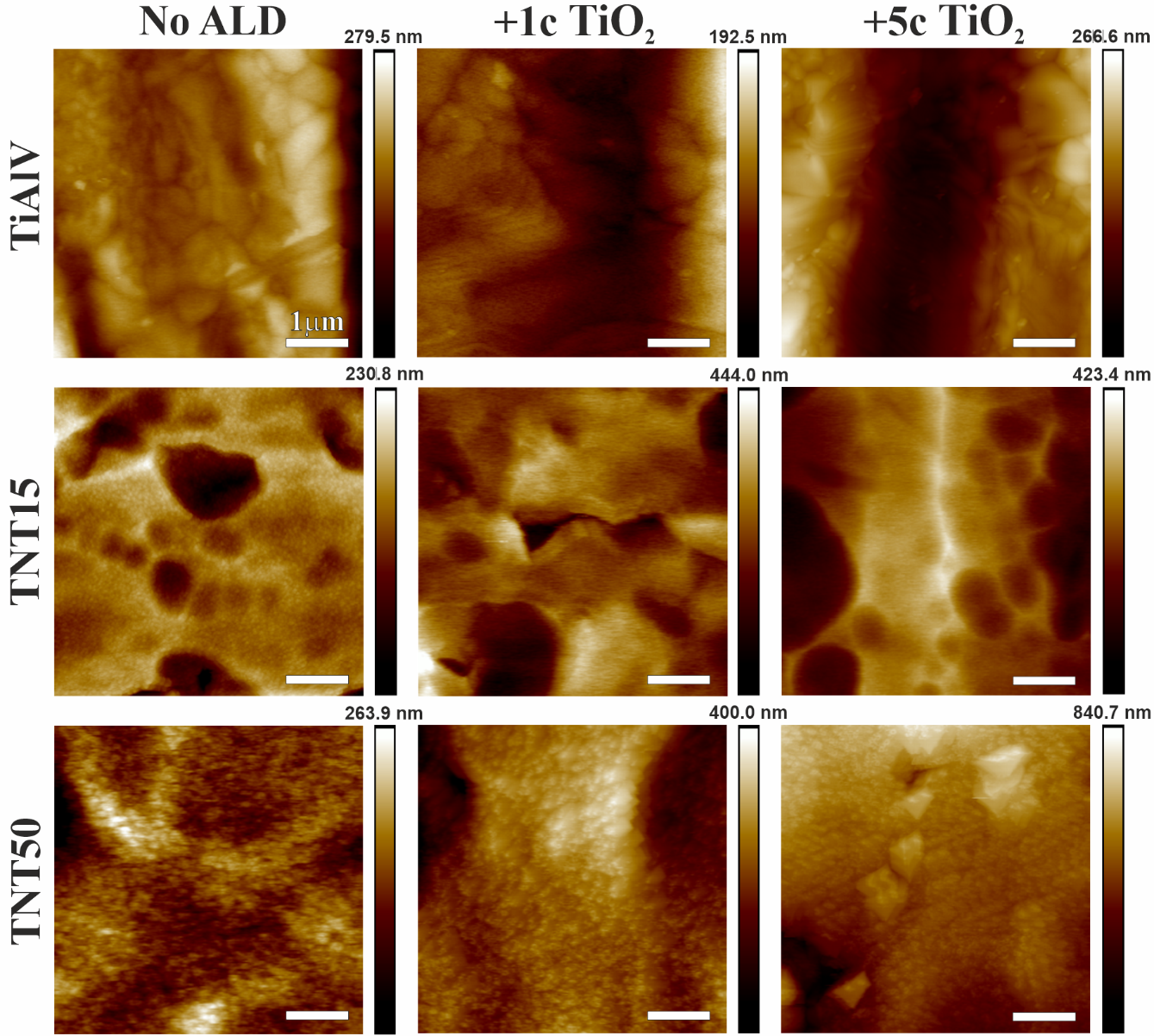


**Figure S4.** AFM topological images of the TiAlV foils, TNT15 and TNT50 layers before and after 1c and 5c TiO_2_ ALD coating.


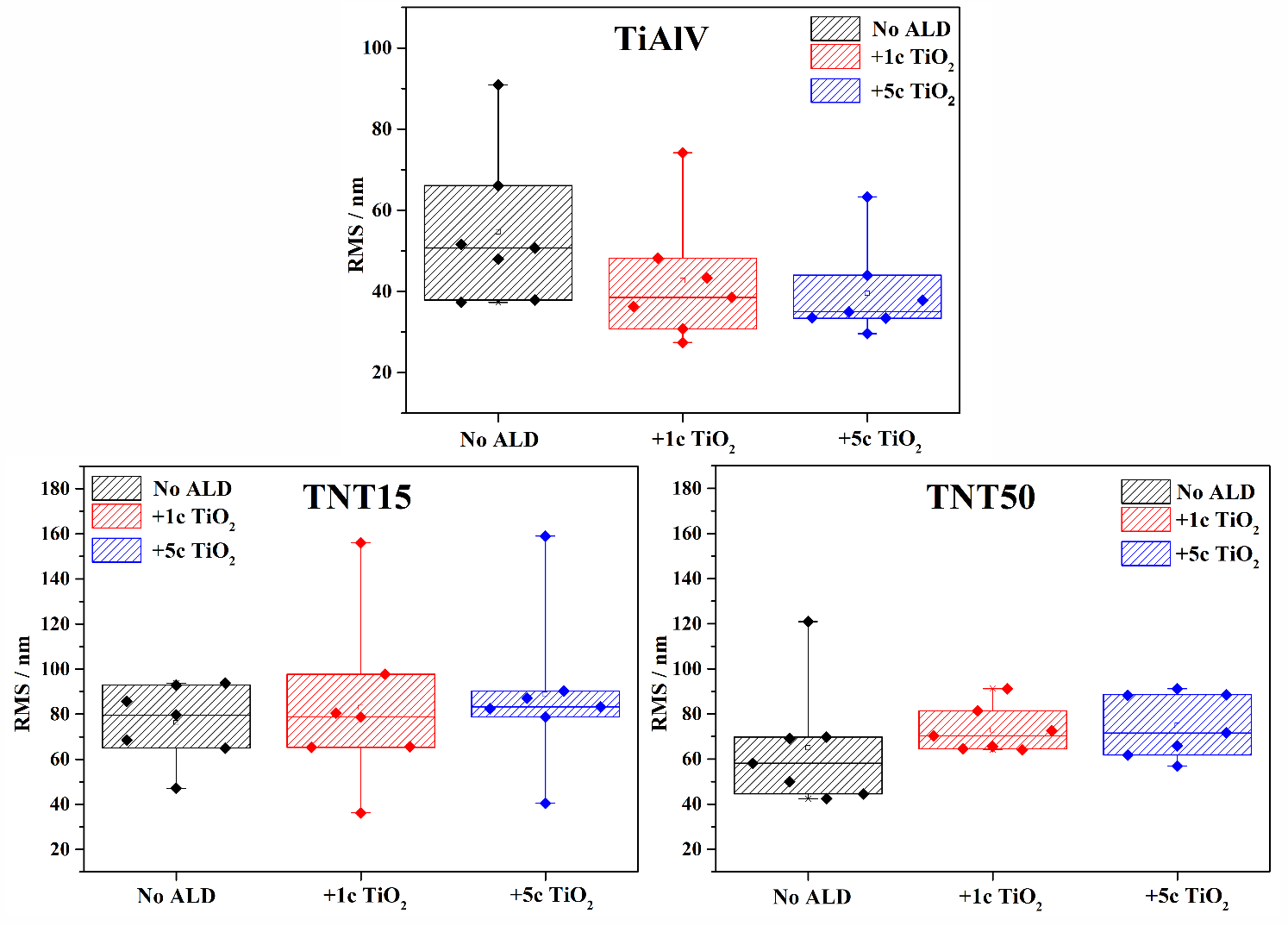


**Figure S5.** Roughness values (root mean square, RMS) obtained by AFM in the form of the box-plot describing mean (open square); 1^st^ and 3^rd^ quantile (box); and min/max values (whisker) of the TiAlV foils, TNT15 and TNT50 layers before and after 1c and 5c TiO_2_ ALD coating.


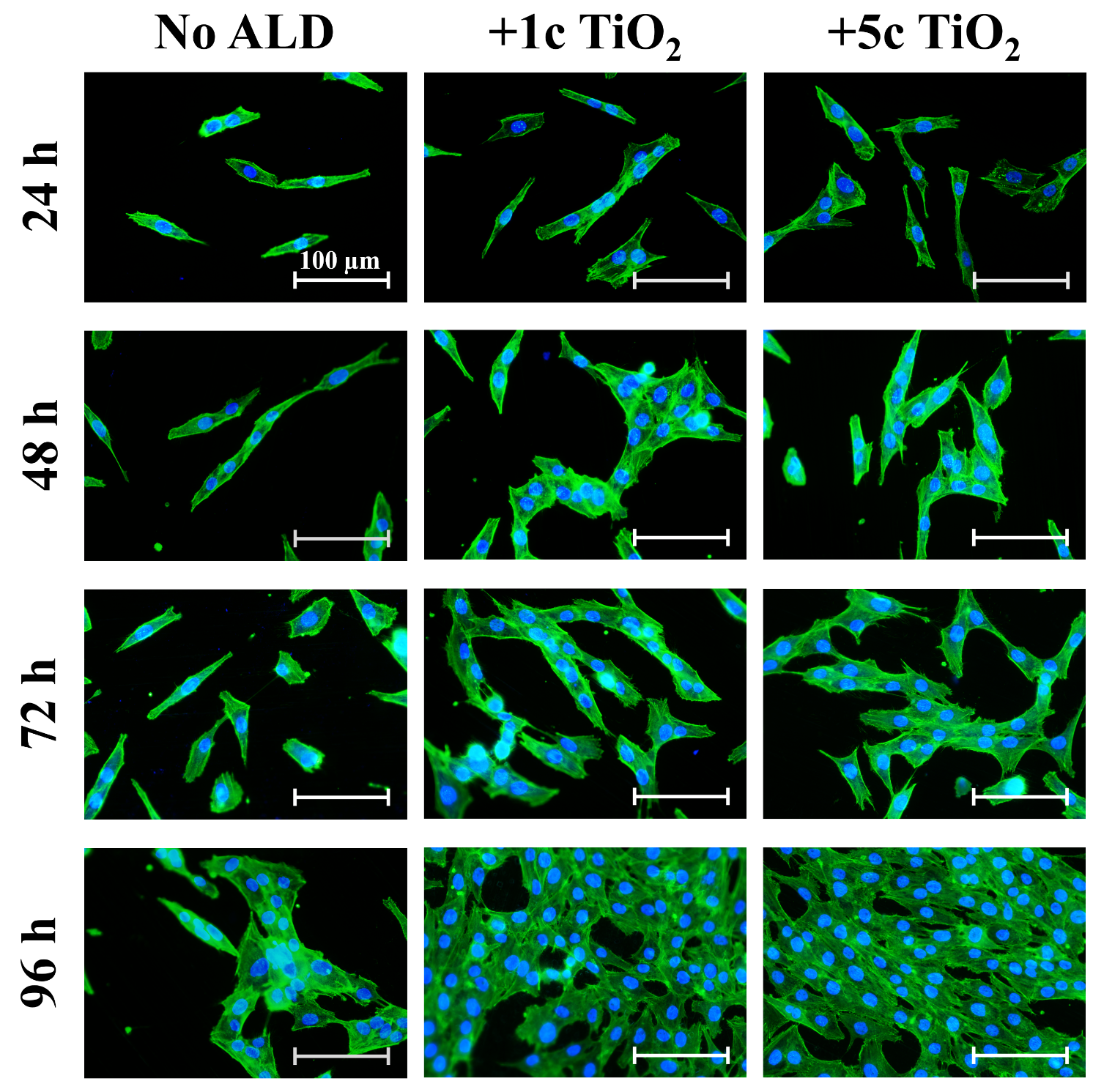


**Figure S6.** Photomicrographs of MG-63 cells grown on uncoated or 1c and 5c TiO_2_ ALD-coated TiAlV foils for 24-96 h (No ALD = without TiO_2_ ALD coating; +1c ALD = 1c TiO_2_ ALD coating; +5c ALD = 5c TiO_2_ ALD coating). The actin filaments were stained with the Phalloidin-FITC probe (green), and the cell´s nuclei were stained with the Hoechst 33258 probe (blue).


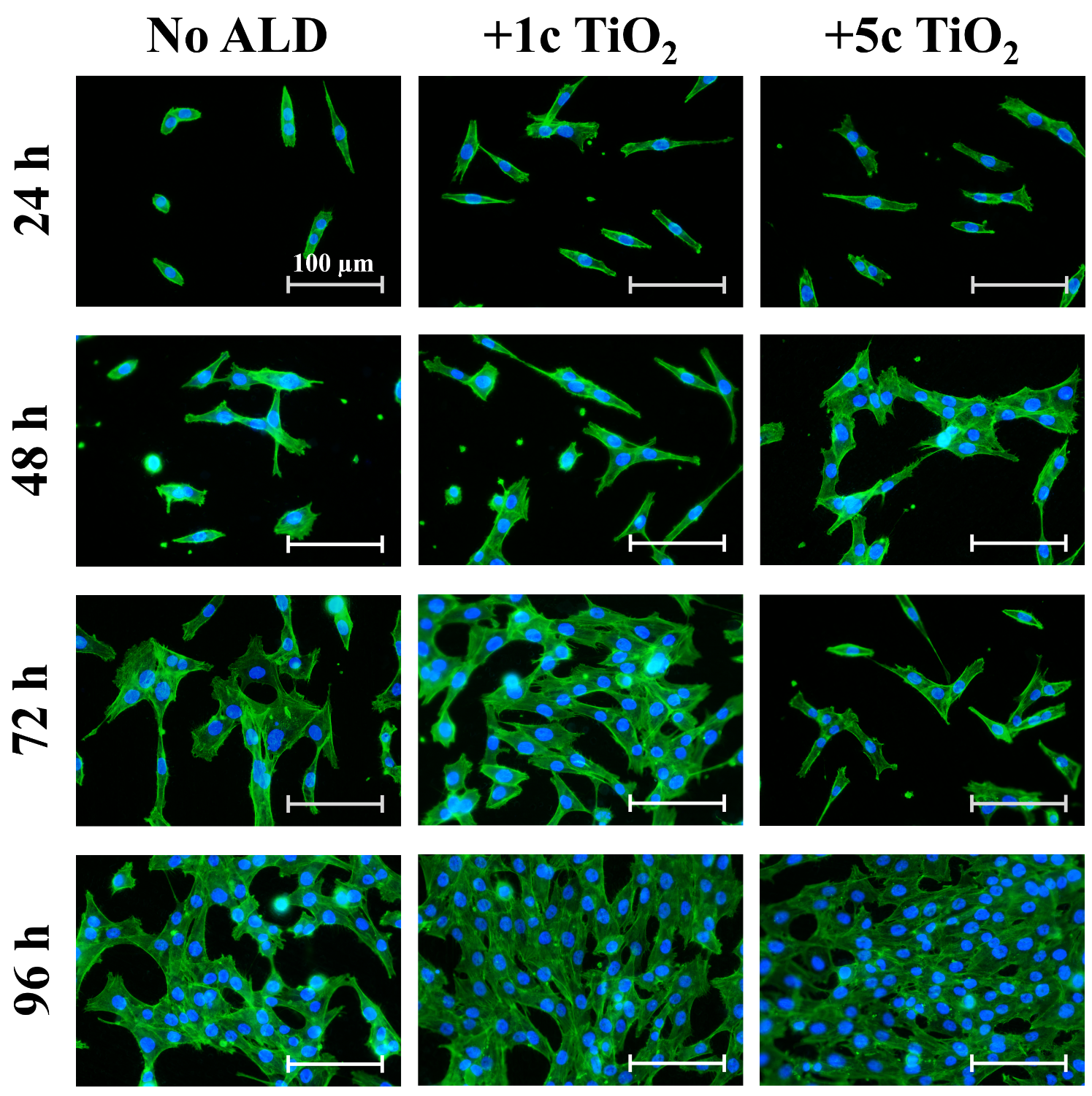


**Figure S7.** Photomicrographs of MG-63 cells grown on uncoated or 1c and 5c TiO_2_ ALD-coated TNT15 layers for 24-96 h (No ALD = without TiO_2_ ALD coating; +1c ALD = 1c TiO_2_ ALD coating; +5c ALD = 5c TiO_2_ ALD coating). The actin filaments were stained with the Phalloidin-FITC probe (green), and the cell´s nuclei were stained with the Hoechst 33258 probe (blue).


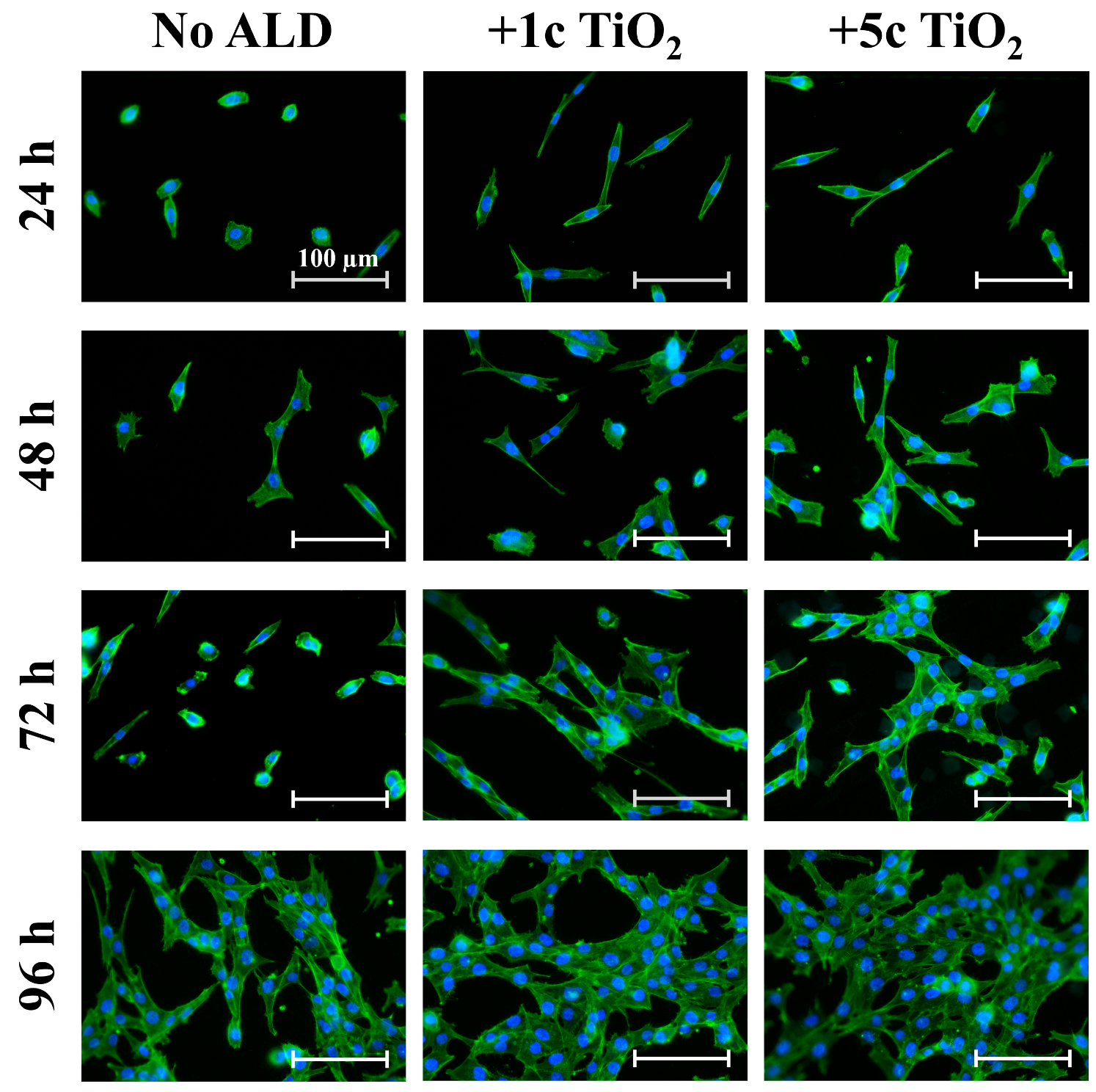


**Figure S8.** Photomicrographs of MG-63 cells grown on uncoated or 1c and 5c TiO_2_ ALD-coated TNT50 layers for 24-96 h (No ALD = without TiO_2_ ALD coating; +1c ALD = 1c TiO_2_ ALD coating; +5c ALD = 5c TiO_2_ ALD coating). The actin filaments were stained with the Phalloidin-FITC probe (green), and the cell´s nuclei were stained with the Hoechst 33258 probe (blue).





**Figure S9.** Analysis of elongation of MG-63 cells grown on uncoated or 1c and 5c TiO_2_ ALD-coated TiAlV foils, TNT15 and TNT50 layers for 24 and 48 h (0 = without TiO_2_ ALD coating; 1 = +1c TiO_2_ ALD coating; 5 = +5c TiO_2_ ALD coating). Data originated from three independent experiments presented as mean ± SEM (**, p<0.01; ***, p < 0.001, vs. 0c at appropriate time interval).
